# Lack of Association between Cervical Spine Injuries and Prehospital Immobilization: From Tradition to Evidence

**DOI:** 10.3390/jcm13164868

**Published:** 2024-08-18

**Authors:** Ilan Y. Mitchnik, Yael V. Ezra, Irina Radomislensky, Tomer Talmy, Ran Ankory, Avi Benov, Shaul Gelikas

**Affiliations:** 1 Medical Corps, Israel Defense Force, Tel HaShomer, Tel Aviv 5510802, Israelsgelikas@gmail.com (S.G.); 2 Department of Military Medicine, Faculty of Medicine, The Hebrew University of Jerusalem, Jerusalem 9190500, Israel; 3 Division of Orthopedic Surgery, Shamir Medical Center, Zrifin 6093000, Israel; 4 Faculty of Medicine, Tel Aviv University, Tel Aviv 6997801, Israel; 5 Trauma and Combat Medicine Branch, Medical Corps, Israel Defense Force, Tel HaShomer, Tel Aviv 5510802, Israel; 6 The National Center for Trauma & Emergency Medicine Research, Gertner Institute for Epidemiology and Health Policy Research, Sheba Medical Center, Tel HaShomer, Tel Aviv 5262160, Israel; 7 Division of Orthopaedic Surgery, Ichilov Medical Center, Tel Aviv 6997801, Israel; 8 Division of Internal Medicine, Sheba Medical Center, Tel HaShomer, Tel Aviv 5262000, Israel

**Keywords:** cervical spine, trauma, prehospital immobilization, clinical practice guidelines

## Abstract

**Background:** Cervical spine (C-spine) trauma usually results from blunt injuries and is traditionally managed by prehospital spinal immobilization using a cervical collar. We sought to examine if prehospital C-spine immobilization is associated with actual C-spine injuries and what factors are associated with the decision to immobilize the C-spine. **Methods:** We retrospectively analyzed blunt trauma patients treated by Israeli Defense Force (IDF) medical teams from 2015 to 2020. Children, penetrating injuries, and non-threatening injuries were excluded. Demographic data, injury characteristics, and prehospital information were collected from the IDF Trauma Registry’s electronic medical records and merged with corresponding hospital data from the Israeli National Trauma Registry. **Results:** Overall, 220 patients were included, with a mean age of 32 and a predominance of male patients (78%). Most injuries were due to motor vehicle collisions (77%). In total, 40% of the patients received a cervical collar. C-spine injuries were present in 8%, of which 50% were immobilized with a cervical collar. There were no significant differences in the incidences of C-spine injuries or disability outcomes with or without collar immobilization. The use of a collar was significantly associated with backboard immobilization (OR = 14.5, *p* < 0.001) and oxygen use (OR = 2.5, *p* = 0.032). **Conclusions:** Prehospital C-spine immobilization was not associated with C-spine injury or neurological disability incidences. C-spine immobilization by medical providers may be influenced by factors other than the suspected presence of a C-spine injury, such as the use of a backboard. Clear clinical guidelines for inexperienced medical providers are called for.

## 1. Introduction

Traumatic injuries are a leading cause of death in developed countries [1]. Spinal trauma, constituting 1–10% of all trauma cases [1,2], is usually caused by blunt trauma, with motor vehicle collisions (MVCs) and falls being common causes [3,4]. Spinal immobilization has been practiced since the 1960s, with the belief that trauma victims with spinal injuries may suffer further neurologic injury if moved without stabilization [5]. Approximately 25% of spinal cord injuries occur during prehospital and early hospital care stages [6]. Therefore, early identification and management of potential major spinal trauma at the injury site are crucial.

Traditionally, spinal immobilization utilizes a rigid backboard and a cervical collar. Properly sized collars limit flexion by up to 90% and extension and lateral bending by up to 50% [7]. Cervical spine (C-spine) collars are believed to reduce compressive forces across the C6, C7, and T1 vertebrae [7]. In theory, cervical collars should protect patients from secondary spinal cord injuries by restricting the movement of unstable cervical spine fractures and other injuries. However, as reviewed by Sundstrøm et al., there is no significant evidence to support this theory [6]. Penetrating trauma, comprising 13% of spinal injuries [8], is exempt from this practice. Penetrating thoracolumbar spine (TL-spine) injuries are deemed to be stable [9,10], while penetrating C-spine injuries are typically fatal onsite [11]. Overall, spinal immobilization for penetrating trauma is discouraged and linked to higher mortality rates [12]. 

Research not only indicates limited clinical benefits to spinal and cervical immobilizations [13], but some studies have also identified significant disadvantages. Backboard immobilization may lead to respiratory compromise [14], and both spinal and cervical immobilizations can result in challenges with airway management [15], pain [16], unnecessary radiological imaging [17], and pressure ulcers [18]. Cervical immobilization can also be complicated by increased intracranial pressure (ICP) due to restricted venous drainage [19]. Although a recent study suggested that soft cervical collars may eliminate this particular complication, soft collars may not adequately stabilize the C-spine [20]. The potential harms of C-spine immobilization are not widely acknowledged, and research suggests that many patients without C-spine injuries still receive cervical collars [21]. In fact, it is estimated that for every significant C-spine injury immobilized, from 50 to 100 patients without injury also undergo immobilization [21].

The gold standard for diagnosing spinal injuries is imaging, but this is, of course, not feasible in prehospital field care. Validated criteria, like those of the national emergency X-radiography utilization study (NEXUS) and the Canadian C-spine rule, may exclude immobilization or imaging requirements in the trauma bay for C-spine injuries [22]. In 2019, an expert panel from the Israeli Defense Force’s Medical Corps (IDF-MC) recommended changes in clinical practice guidelines (CPGs) for spinal immobilization. The panel discouraged rigid-backboard use, which is advocated by prehospital trauma life-support (PHTLS) doctrines, because of its drawbacks and insufficient evidence supporting its benefits. Instead, the panel proposed a modified technique using simple fabric stretchers, which theoretically have more contact area with the spine. A recommendation was also made to continue the use of cervical collars for suspected C-spine injuries, pending further research. Thus, this work seeks to answer two questions regarding the practices of prehospital C-spine immobilization: (1) Is prehospital C-spine immobilization correlated with actual C-spine injuries? and (2) What factors are associated with the decision to immobilize the C-spine in the prehospital setting?

## 2. Materials and Methods

### 2.1. Study Design and Participants

This retrospective study analyzed trauma victims treated by IDF-MC advanced life-support (ALS) providers in the prehospital setting between 1 January 2015 and 31 December 2020. Data were collected from the IDF Trauma Registry’s (IDF-TR’s) electronic medical records and merged with hospital data from the Israeli National Trauma Registry (INTR). The merging process excluded prehospital fatalities and injured patients not hospitalized in Israeli hospitals. This study was approved by an IDF-MC institutional review board (approval no. 1948–2018).

This study included patients, treated by IDF ALS medical teams, who sustained injuries from falls or motor vehicle collisions (MVCs). Exclusions comprised patients lacking documentation of injured body regions, those with documented penetrating injuries, individuals categorized as “non-urgent” onsite (no apparent risk to life, end organs, or limbs), patients under the age of 14 years (because of anatomical and physiological differences), cases labeled with “strike by an object” as the primary injury mechanism (given its broad usage for various mechanisms), cases lacking documentation of the ALS provider (to account for potential training differences), and cases without a documented diagnosis of C-spine injury.

### 2.2. IDF Medical Teams

The training of the ALS providers within the IDF-MC varies depending on their roles, with doctors and paramedics undergoing distinct training programs [23]. Doctors primarily receive professional training in hospitals and subsequently undergo IDF Military Trauma Life-Support (MTLS) training to follow the IDF Trauma and Combat Medicine Branch (TCMB) CPGs upon enlistment. On the other hand, paramedics initially train with Magen David Adom (MDA), the national civilian emergency medical service, following PHTLS guidelines. After completing paramedic training, they undergo the same IDF MTLS course. Notably, military paramedics maintain operational competence by completing a minimum of four competence shifts monthly in MDA ambulances. Similarly, physicians must fulfill at least two operating room shifts to ensure ongoing trauma treatment competence. IDF medical teams are frequently called to assist in civilian medical emergencies, and it is not uncommon for both MDA and IDF medical teams to be present at the same location.

### 2.3. Cervical Injury CPGs

**Figure 1:**
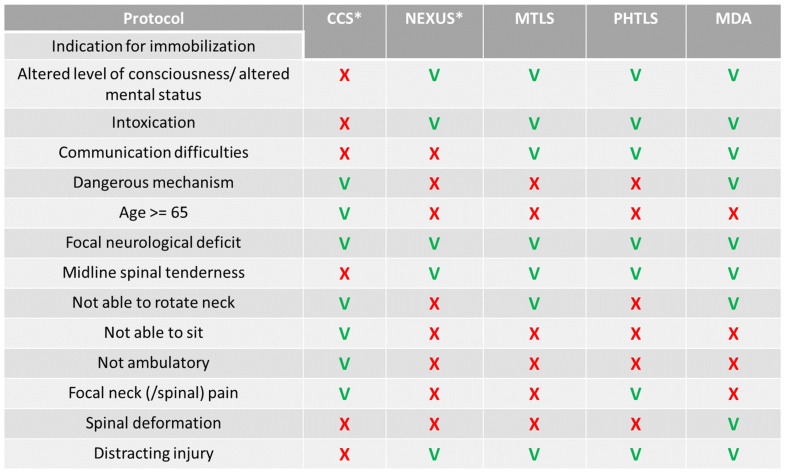
Comparison of cervical spine management protocols. CCS = Canadian cervical spine rule; NEXUS = national emergency X-radiography utilization study; MTLS = military trauma life-support protocols of the Israeli Defense Forces; PHTLS = prehospital life-support protocols; MDA = Magen David Adom (the Israeli civilian organization for prehospital care) protocols. (*) The CCS and the NEXUS criteria are meant to indicate spinal imaging in-hospital; the MTLS, PHTLS, and MDA protocols are meant to indicate spinal immobilization in the prehospital setting.

The IDF MTLS protocols share common criteria with NEXUS [22]. These recommend that a cervical collar should not be used for patients who are fully alert, communicative, not intoxicated, without focal neurological deficits or other distracting injuries, and who can move their neck freely without focal posterior midline cervical spine tenderness. This is different from PHTLS guidelines, which also mandate immobilization when the patient has a substantial head injury [7]. MDA CPGs further recommend immobilization practices when the injury mechanism involves severe kinematics and when a spinal deformity is present (Figure 1).

### 2.4. Data Collection

We collected demographic data, encompassing age, gender, and injury information, including the mechanism and the presence of C-spine and TL-spine injuries and other musculoskeletal injuries, as recorded using the abbreviated injury scale (AIS). Data about the injury setting included the event type—military circumstances (trauma during military operations or training) and non-military circumstances (all other situations)—ALS provider type (doctor only, paramedic only, or both), and the number of casualties. Prehospital victim status data included AVPU scores (a basic consciousness scale—declaring the patient’s state as being alert, responsive to vocal stimulation, responsive to painful stimulation, or unresponsive), Glasgow coma scale (GCS) scores, pulse oximetry readings (SPO2), hemodynamic status—including the hemodynamic shock state (defined as a systolic blood pressure of <90 mm/hg)—and pain scores (assessed using the visual analog scale, VAS). In addition, we collected data for prehospital medical procedures, such as oxygen supplementation, endotracheal intubation (ETI), cricothyroidotomy, needle decompression, chest drain insertion, backboard immobilization, and cervical collar application. 

### 2.5. Statistical Analysis

Patients were divided into two main groups: the collar group (patients with documentation of cervical collar application in a designated rubric or free text in the report file) and the no-collar group (patients without such documentation or with clear documentation that a cervical collar was not applied). Patients were also divided into two other groups: the C-spine-injury group (patients with documentation of a C-spine injury diagnosed during their subsequent hospitalization according to the INTR) and the no-C-spine-injury group (patients without such documentation).

Descriptive statistics were used to summarize the demographic and injury characteristics of the study sample. Chi-squared and Fisher’s exact tests were used to compare differences between groups for categorical variables, and Mann–Whitney U was used to compare differences between groups for continuous variables. Multivariable logistic regression was used to assess factors associated with the use of cervical collars. The multicollinearity of the variables included in the model was evaluated using the variance inflation factor (VIF) with a cutoff value of 5. A *p*-value of <0.05 was considered as statistically significant. All the analyses were performed by a statistician using R software, version 4.0.3 (R Foundation for Statistical Computing, Vienna, Austria).

## 3. Results

### 3.1. Patient and Injury Characteristics 

Out of 1000 trauma cases identified, we excluded 387 cases with mechanisms other than a fall or an MVC, 15 with a penetrating injury, 26 pediatric cases, 297 non-urgent cases, and 55 poorly documented cases (no known ALS provider or no diagnosis). Overall, 220 patients were included for the analysis, with 87 (39.5%) receiving a cervical collar (Table 1). Most patients were male (77.7%), with a median age of 22.50 (interquartile range = 20 to 35). The primary injury mechanism was an MVC (76.8%, Table 1). Of the 220 patients, 18 (8.2%) had a C-spine injury (Table 2). Patients with a C-spine injury had significantly more TL-spine injuries and thoracic injuries compared to those without (55.6% compared to 18.8%, *p* = 0.001 and 66.7% compared to 39.6%, *p* = 0.043, respectively; Table 2). No other significant demographic difference was found between patients with and without C-spine injuries (Supplemental Table S1).

Out of the 18 patients in the C-spine-injury group, 9/18 (50%) had a cervical collar applied, while the other 9/18 (50%) did not. As shown in Table 2, upon hospital discharge, 5/18 (27.8%) of the C-spine-injury group required rehabilitation, 11/18 (61.1%) were discharged to other care, and 2/18 (11.1%) died during hospitalization. The disability rate in the C-spine-injury group was insignificantly higher compared to that in the group with no C-spine injury (3/16 (18.8%) compared to 34/199 (17.1%), *p* = 0.057; Table 2). There were no significant differences in the prevalences of hemiplegia, paraplegia, and tetraplegia between the C-spine-injury and no-C-spine-injury groups (Table 2).

### 3.2. Cervical Collar Use and Cervical Spine Injuries

As shown in Table 1, the incidences of C-spine injury were similar between the collar group and the no-collar group (10.3% and 6.8%, respectively, *p* = 0.451). Hospital discharge outcomes and disability rates also did not differ significantly between the two groups, with the majority in both groups experiencing no disability (Table 1). Although it was not statistically significant, the only notable difference between the groups was the type of disability, with higher proportions of those in the collar group experiencing chronic pain, limited ROM, or spinal fixation as a disability (9.2% compared to 3.8%, *p* = 0.142; Table 1).

### 3.3. Prehospital Treatment and Factors Associated with Cervical Collar Use

Most events (61.1%) were attended by both doctors and paramedics, with 49.5% being a single casualty event and the rest being multi-casualty events (Table 3). There was no significant difference in the event type (military or non-military circumstances) between the two groups of the collar application (Table 3); however, there was a significant difference in the victim status, with a higher proportion of patients treated with a collar having lower consciousness levels, with GCS scores below 14 (28.7% compared to 17.3%, *p* = 0.038; Table 3). Hemodynamic status also significantly differed between the groups, with a higher proportion of patients treated with a collar suffering from shock (9.2% compared to 4.5%, *p* = 0.007; Table 3). There were no significant differences in the event scenario and other measures of victim status between the two groups (Table 3). Collar use was significantly associated with backboard immobilization (*p* < 0.001), with a higher percentage of the collar group (66.7%) immobilized with a backboard compared to the no-collar group (15.8%). No significant association was shown with the other medical procedures, as described in Table 3. 

The adjusted odds ratios (AORs) for collar use and victim type were significantly higher for soldiers, at 2.90, with 95% confidence intervals [95% CIs] from 1.04 to 8.40. The AORs for collar use and interventions showed that the use of oxygen had an AOR of 2.52 [95% CI, from 1.10 to 5.95], and the use of a backboard had an AOR of 14.52 [95% CI, from 6.96 to 32.41]. All the other associations between collar use and the other variables, including GCS scores and hemodynamic status, were not statistically significant (Supplemental Table S2).

## 4. Discussion

The most important findings in our study are the lack of a significant association between prehospital C-spine immobilization and actual C-spine injuries or patient outcomes, such as neurological disabilities or disability rates. Our second finding is that the decision to immobilize the C-spine in the prehospital setting appears to be associated with factors other than the suspected presence of a C-spine injury—such as the use of a backboard and oxygen supplementation. We also found that the use of a prehospital cervical collar was not significantly associated with airway and breathing interventions, such as endotracheal intubation, coniotomy, needle decompression, or chest drain insertions.

The results of our study shed light on prehospital C-spine immobilization practices, indicating from limited to no impact on patient outcomes after C-spine trauma. These findings align with those of prior studies that have questioned the benefits of spinal immobilization in the prehospital setting [13,14,15,16,17,18]. Castro-Mari et al. even showed that reducing the number of backboard immobilizations did not increase the incidence of spinal cord injuries [24]. Although our study revealed similar C-spine injury rates (8.2%) compared to other studies [1,2], a noteworthy 50% of the C-spine injuries did not receive a cervical collar at the injury site, suggesting potential oversights and limitations of PHTLS (or MDA) and MTLS (or NEXUS) CPGs. To the best of our knowledge, this is the first study reporting a missed-C-spine-injury rate in the prehospital setting, raising concerns about the efficacy of current CPGs in detecting spinal injuries. It is important to note that although there was no significant difference in outcomes between the collar and no-collar groups, the subgroup of patients with C-spine injuries had significantly worse outcomes compared to the no-C-spine-injury group, including higher mortality rates (11.1% compared to 1.5%, Table 2) and a greater need for rehabilitation (27.8% compared to 19.3%, Table 2). These findings should stress the importance for recognizing these injuries, and if collars truly protect the spine as traditionally believed then a 50% missing rate by CPGs is concerning.

Despite the increased morbidity and mortality associated with cervical spine injuries, our study did not find significantly higher rates of neurological disabilities in patients with cervical spine injuries (Table 2). One possible explanation is some of the victims suffered fatal spinal cord injuries. Another explanation is a relatively small sample size. Hasler et al. [1] described the rate of spinal cord injuries in patients with spine injuries as being 1.8%. Given the prevalence of 8.2% cervical spine injuries in our study, we would expect 0.1% to have a spinal cord injury—which could either be transient or chronic. Within our cohort of 18 cervical spine injuries, we detected 0% with spinal cord injuries. This finding further highlights the rare incidence of non-fatal spinal cord injuries in young patients with cervical spine injuries. Whether a cervical collar protects these patients from such rare neurological injuries cannot be ascertained with this study design and sample size.

In our study, patients immobilized with a backboard, those requiring oxygen support, and those who were soldiers were more likely to receive cervical collars. Our observations suggest that IDF medical teams tend to apply cervical collars to patients who are already immobilized by backboards. This is possibly because of PHTLS (and MDA) CPGs recommending backboard use for suspected spine injuries, which share similar indications with cervical spine injuries (Figure 1). As described in the introduction, in 2019, an IDF-MC expert panel reviewed the evidence regarding the use of backboard immobilizations. This panel did not find sufficient evidence to support the potential benefits of backboard immobilization against its negative effects, including respiratory compromise, pressure ulcers, and pain. Instead, the panel suggested the use of simple canvas stretchers, which provide more contact surface with the body than rigid backboards. These recommendations were not yet put into effect prior to 2020, when this study period took place. Thus, during the study period, the usage of rigid backboards was still practiced by IDF medical teams. Our concern is that in cases where a backboard was used to extricate trapped victims, the presence of the backboard itself may have convinced ALS providers to also place a collar despite no clear indication for spinal injury. Unfortunately, such immobilization practices provide a false sense of stabilization security [7].

Our observations show that patients with cervical immobilizations were more likely to require oxygen supplementation without airway or breathing interventions (such as ETIs, chest drain insertion, or needle decompression, Table 3). The underlying reasons for this association are not completely explained by our study because C-spine injuries were not associated with airway or breathing interventions (Supplemental Table S2). Two possible explanations are that these patients also had lower consciousness levels (Table 3) or that cervical immobilization caused respiratory compromise because of a reduced effective lung capacity [14]. This practice was also shown to be associated with difficulty in airway management interventions (specifically chin lifts and ETIs) [15]. We also observed that cervical collars were more commonly applied to patients in hemodynamic shock (Table 3); however, this was insignificant upon regression analysis (Supplemental Table S2). A similar finding was also reported by Häske et al. [2] and may indicate that hemodynamic shock is due to some distracting injury, such as exsanguinating bleeding; however, because the association between hemodynamic shock and collar application is not reflected in the regression model, we may also assume that hemodynamic shock indicates a highly kinematic injury, which, in turn, correlates with the choice to apply a collar because of the suspected mechanism of high-energy blunt injury (Figure 1).

The CPGs for the management of C-spine injuries in the IDF during the studied period may have been insufficient, despite uniform MTLS training with CPGs similar to NEXUS and the PHTLS (Figure 1). These CPGs also may not be effective—both in detecting potential C-spine injuries and in preventing their complications. Thus, there is a need to invest efforts into the implementation of clear and consistent protocols, especially for inexperienced prehospital ALS providers. This has led us to develop a new, uniform, CPG for IDF MTLS training (see Supplemental Figure S1). Future research will validate whether this CPG improves C-spine injury detection and reduces complication rates.

Our research has certain limitations that need to be acknowledged. One of these is reporting bias in the prehospital setting, which means that not all the instances of cervical collar application may have been recorded. This bias also appears in some missing data (mainly for VAS levels), as shown in Table 3. Additionally, information on disabilities was only available for patients who applied for rehabilitation, which may have resulted in some further reporting bias. Another potential limitation is selection bias, given that a large proportion of our study participants were soldiers, who are typically young and in good health. Moreover, we only included patients who arrived at the hospital alive, which means that our findings may not generalize to those with more fatal injuries onsite. Finally, the retrospective nature of our study precludes us from discerning the exact CPG that each medical provider adhered to.

## 5. Conclusions

In conclusion, prehospital C-spine immobilization was not associated with reduced C-spine injury rates or neurological disability rates. The decision to immobilize the C-spine in the prehospital setting was influenced by factors such as the use of backboard immobilization and the need for oxygen supplementation. Prehospital cervical immobilization practices missed many injuries, and although a lack of cervical immobilization for true cervical spine injuries may not have affected their prognosis, more accurate CPGs are still called for. These CPGs should assert proper clinical judgment and not rely simply on the use of a backboard for victim extrication, while also considering the potential effects of cervical collars on oxygenation requirements.

## Figures and Tables

**Table 1:** Patient demographics and cervical collar outcomes.

	Collar (N = 87)	No Collar (N = 133)	*p*-Value
**Patient Demographics**			
**Age**			0.537
Mean (SD)	32.43 (15.90)	31.07 (15.36)	
**Gender, N (%)**			0.249
Female	23 (26.4%)	26 (19.5%)	
Male	64 (73.6%)	107 (80.5%)	
**Casualty Population, N (%)**			0.390
Soldier	28 (32.2%)	51 (38.3%)	
Not Soldier	59 (67.8%)	82 (61.7%)	
**Event Type, N (%)**			0.126
Military Circumstances	9 (10.3%)	25 (18.8%)	
Non-military Circumstances	78 (89.7%)	108 (81.2%)	
**Injury Mechanism, N (%)**			0.625
MVC	65 (74.7%)	104 (78.2%)	
Fall	22 (25.3%)	29 (21.8%)	
**Patient Outcomes**			
**Cervical Injury Present, N (%)**	9 (10.3%)	9 (6.8%)	0.451
**Hospital Discharge, N (%)**			0.751
Death	2 (2.3%)	3 (2.3%)	
Rehabilitation	15 (17.2%)	29 (21.8%)	
Other	70 (80.5%)	101 (75.9%)	
**Disability Rate, N (%)**			0.618
None	72 (82.8%)	106 (79.7%)	
0–19%	2 (2.3%)	4 (3.0%)	
20–49%	8 (9.2%)	9 (6.8%)	
50–99%	2 (2.3%)	10 (7.5%)	
100%	1 (1.1%)	1 (0.8%)	
Died	2 (2.3%)	3 (2.3%)	
**Disability Type, N (%)**			
Cervical Spine: Chronic Pain, Limited ROM, or Spinal Fixation	8 (9.2%)	5 (3.8%)	0.142
Hemiplegia	2 (2.3%)	2 (1.5%)	0.649
Paraplegia	0 (0.0%)	1 (0.8%)	1.000
Tetraplegia	0 (0.0%)	0 (0.0%)	1.000

This table compares the demographic and injury characteristics of patients with suspected cervical spine blunt trauma treated with or without cervical collars by prehospital medical teams. The table also describes these patients’ outcomes following evacuation for hospitalization. N = number; SD = standard deviation; % = valid percentage; Collar = patients with documentation of cervical collar application in the designated rubric or in free text in the report file; No Collar = patients without documentation of collar application or with clear documentation that a cervical collar was not applied; Military Circumstances = events including either trauma sustained during military operations or military training; Non-military Circumstances = events that are not related to military operations or training; MVC = motor vehicle collision; ROM = range of motion.

**Table 2:** Cervical spine injuries and outcomes.

	C-Spine Injury (N = 18)	No C-Spine Injury (N = 202)	*p*-Value
**Body Region, N (%)**			
TL-spine	10 (55.6%)	38 (18.8%)	**0.001**
Head	10 (55.6%)	82 (40.6%)	0.225
Neck	0 (0.0%)	5 (2.5%)	1.000
External	0 (0.0%)	3 (1.5%)	1.000
Thorax	12 (66.7%)	80 (39.6%)	**0.043**
Abdomen	8 (44.4%)	60 (29.7%)	0.196
Face	9 (50.0%)	53 (26.2%)	0.052
Upper Extremity	8 (44.4%)	74 (36.6%)	0.612
Lower Extremity	7 (38.9%)	86 (42.6%)	0.259
**Hospital Discharge, N (%)**			**0.031**
Death	2 (11.1%)	3 (1.5%)	
Rehabilitation	5 (27.8%)	39 (19.3%)	
Other	11 (61.1%)	160 (79.2%)	
**Disability Rate, N (%)**			0.057
None	13 (72.2%)	165 (81.7%)	
0–19%	0 (0.0%)	6 (3.0%)	
20–49%	3 (16.7%)	14 (6.9%)	
50–99%	0 (0.0%)	12 (5.9%)	
100%	0 (0.0%)	2 (1.0%)	
Died	2 (11.1%)	3 (1.5%)	
**Disability Type, N (%)**			
Cervical Spine: Chronic Pain, Limited ROM, or Spinal Fixation	3 (16.7%)	10 (5.0%)	0.078
Hemiplegia	0 (0.0%)	4 (2.0%)	1.000
Paraplegia	0 (0.0%)	1 (0.5%)	1.000
Tetraplegia	0 (0.0%)	0 (0.0%)	

This table compares the injury patterns and outcomes of patients with and without confirmed cervical spine (C-spine) injuries. Bold = statistically significant *p*-value; N = number; C-spine = cervical spine; TL-spine = thoracolumbar spine; ROM = range of motion.

**Table 3:** Patient status at the prehospital stage.

	Collar (N = 87)	No Collar (N = 133)	*p*-Value
**ALS Provider, N (%)**			0.211
Doctor Only	7 (8.0%)	17 (12.8%)	
Paramedic Only	36 (41.4%)	64 (48.1%)	
Doctor and Paramedic Together	44 (50.6%)	52 (39.1%)	
**Number of Casualties, N (%)**			0.531
1	46 (52.9%)	63 (47.4%)	
2–3	19 (21.8%)	27 (20.3%)	
4+	22 (25.3%)	43 (32.3%)	
**AVPU, N (%)**			0.467
A	54 (62.1%)	90 (67.7%)	
V	6 (6.9%)	7 (5.3%)	
P	7 (8.0%)	9 (6.8%)	
U	13 (14.9%)	11 (8.3%)	
Missing Data	7 (8.1%)	16 (11.9%)	
**GCS, N (%)**			**0.038**
3–8	17 (19.5%)	15 (11.3%)	
9–13	8 (9.2%)	8 (6.0%)	
14–15	57 (65.5%)	88 (66.2%)	
Missing Data	5 (5.8%)	22 (16.5%)	
**Pain (VAS), N (%)**			0.961
0	1 (1.1%)	1 (0.8%)	
1–4	5 (5.7%)	6 (4.5%)	
5–10	40 (46.0%)	61 (45.9%)	
Missing Data	41 (47.2%)	65 (48.8%)	
**Hemodynamic Status, N (%)**			**0.007**
Shock	8 (9.2%)	6 (4.5%)	
No Shock	78 (89.7%)	113 (85.0%)	
Missing Data	1 (1.1%)	14 (10.5%)	
**Procedures, N (%)**			
Backboard Immobilization	58 (66.7%)	21 (15.8%)	**<0.001**
Airway Only	1 (1.1%)	0 (0.0%)	0.395
ETI	14 (16.1%)	11 (8.3%)	0.085
Coniotomy	2 (2.3%)	0 (0.0%)	0.155
Needle Decompression	4 (4.6%)	2 (1.5%)	0.216
Chest Drain	1 (1.1%)	0 (0.0%)	0.395

This table compares prehospital scene characteristics, patients’ vital signs, and medical procedures between patients with cervical spine blunt trauma who were treated with or without cervical collar immobilization by prehospital medical teams. Collar = patients with documentation of cervical collar application in the designated rubric or in free text in the report file; No Collar = patients without documentation of collar application or with clear documentation that a cervical collar was not applied; ALS = advanced life support; AVPU = alert, responsive to vocal stimulation, responsive to pain, or unresponsive; GCS = Glasgow coma scale; VAS = visual analog scale (for pain); ETI = endotracheal intubation; Bold = statistically significant *p*-value; N = number; % = valid percentage.

## Data Availability

Data are available upon request but are limited because of IDF Security Intelligence restrictions.

## Supplementary Materials

The following supporting information can be downloaded at https://www.mdpi.com/article/10.3390/jcm13164868/s1: **Supplemental Table S1—Patient procedures.** This table describes the prehospital medical procedures that patients underwent. Patients with a confirmed cervical spine (C-spine) injury are compared to patients without a confirmed C-spine injury. **Supplemental Table S2—Regression models.** This table describes unadjusted and adjusted regression models for potential factors associated with the decision to place cervical collars on patients with blunt cervical trauma. **Supplemental Figure S1—Suggested clinical practice guidelines.** These are suggested clinical practice guidelines (CPGs) for medical providers with limited clinical experience tending to patients with suspected cervical spine injuries in prehospital settings.
