# Supplementary material for: Lack of Association between Cervical Spine Injuries and Prehospital Immobilization: From Tradition to Evidence
Source: J Clin Med. 2024 Aug 18;13(16):4868. doi: 10.3390/jcm13164868 (PMC11355150; doi:10.3390/jcm13164868)
Supplement: Supplementary file 1 [file jcm-13-04868-s001.zip › Supplemental Table S1.pdf]

**Supplemental Table S1 - Patient Procedures:**

| | ***C-spine injury (N = 18)*** | ***No C-spine injury (N = 202)*** | ***Total (N = 220)*** | ***P value*** |
|---|---|---|---|---|
| ***Age*** | | | | 0.151 |
| *Mean (SD)* | 34.11 (13.20) | 31.39 (15.75) | 31.61 (15.55) | |
| *Range* | 18.00 - 64.00 | 14.00 - 81.00 | 14.00 - 81.00 | |
| *Median (IQR)* | 31.50 (23.50 to 41.75) | 25.00 (20.00 to 39.50) | 26.00 (20.00 to 40.00) | |
| ***Age group*** | | | | 0.469 |
| *14-17* | 0 (0.0%) | 15 (7.4%) | 15 (6.8%) | |
| *18-30* | 8 (44.4%) | 109 (54.0%) | 117 (53.2%) | |
| *31-50* | 7 (38.9%) | 53 (26.2%) | 60 (27.3%) | |
| *51+* | 3 (16.7%) | 25 (12.4%) | 28 (12.7%) | |
| ***Gender, N (%)*** | | | | 0.559 |
| *Female* | 5 (27.8%) | 44 (21.8%) | 49 (22.3%) | |
| *Male* | 13 (72.2%) | 158 (78.2%) | 171 (77.7%) | |
| ***Casualty population, N (%)*** | | | | 0.121 |
| *Soldier* | 3 (16.7%) | 76 (37.6%) | 79 (35.9%) | |
| *Not soldier* | 15 (83.3%) | 126 (62.4%) | 141 (64.1%) | |
| ***Event type, N (%)*** | | | | 0.083 |
| *Military circumstances* | 0 (0.0%) | 34 (16.8%) | 34 (15.5%) | |
| *Non-military circumstances* | 18 (100.0%) | 168 (83.2%) | 186 (84.5%) | |
| ***Injury mechanism, N (%)*** | | | | 1.000 |
| *MVC* | 14 (77.8%) | 155 (76.7%) | 169 (76.8%) | |
| *Fall* | 4 (22.2%) | 47 (23.3%) | 51 (23.2%) | |
| ***Procedures, N (%)*** | | | | |
| *Backboard Immobilization* | 9 (50.0%) | 70 (34.7%) | 79 (35.9%) | 0.208 |
| *Airway only* | 0 (0.0%) | 1 (0.5%) | 1 (0.5%) | 1.000 |
| *ETI* | 3 (16.7%) | 22 (10.9%) | 25 (11.4%) | 0.439 |
| *Coniotomy* | 0 (0.0%) | 2 (1.0%) | 0 (0.9%) | 1.000 |
| *Cervical collar* | 9 (50.0%) | 78 (38.6%) | 87 (39.5%) | 0.451 |

This table describes the prehospital medical procedures that patients underwent. Patients with a confirmed cervical spine (c-spine) injury are compared to patients without a confirmed c-spine injury.

**N** = number; **SD** = standard deviation; **IQR** = interquartile range;

**C-spine** = cervical spine; **Military circumstances** = events include either trauma sustained during military operations or military training; **Non-military circumstances** = events that are not related to military operation or training; **MVC** = motor vehicle collision; ***ETI*** *= endotracheal intubation;* ***Coniotomy*** *= cricothyroidotomy;*
