# Supplementary material for: Lack of Association between Cervical Spine Injuries and Prehospital Immobilization: From Tradition to Evidence
Source: J Clin Med. 2024 Aug 18;13(16):4868. doi: 10.3390/jcm13164868 (PMC11355150; doi:10.3390/jcm13164868)
Supplement: Supplementary file 1 [file jcm-13-04868-s001.zip › Supplemental Table S2.pdf]

<u>Supplemental Table S2 – Regression Models:</u>

outcome = collar	*Unadjusted Model*		*Adjusted Model*		
	**OR**	**95% CI**	**AOR**	**95% CI**	**P value**
C-spine Injury	1.59	0.60-4.24	0.98	0.28-3.50	0.974
Spinal injury	2.59	0.84-8.84			
Gender					
Male	0.68	0.36-1.29			
Age					
14-17	1.32	0.38-4.75	3.69	0.75-18.74	0.109
18-30	0.60	0.26-1.34	0.54	0.16-1.85	0.325
31-50	0.88	0.36-2.19	1.35	0.43-4.35	0.61
Victim Type					
Soldier	0.76	0.43-1.34	2.90	1.04-8.40	**0.045**
Event Type					
Military circumstances	0.50	0.21-1.09	0.49	0.14-1.67	0.256
Mechanism of Injury					
MVC	0.82	0.44-1.57	0.87	0.37-2.07	0.757
Number of Casualties					
4+	0.70	0.34-1.32			
2-3	0.96	0.48-1.93			
Intervention					
Oxygen	1.73	0.95-3.16	2.52	1.10-5.95	**0.032**
ETI	2.13	0.92-5.03	5.43	0.66-43.86	0.107
Needle	3.16	0.60-23.14			
Backboard	10.67	5.69-20.74	14.52	6.96-32.41	**<0.001**
GCS					
<9	1.75	0.81-3.82	0.54	0.08-3.28	0.514
9-13	1.54	0.54-4.42	1.38	0.36-5.39	0.635
AVPU					
V	1.43	0.44-4.52			
P	1.30	0.44-3.68			
U	2.00	0.82-4.79			
Pain (VAS)					
1-4	0.83	0.03-24.90			
5-10	0.66	0.03-16.90			
SPO2					
<90	1.38	0.51-3.70	0.91	0.20-4.08	0.897
Hemodynamic Status					
Shock	1.93	0.65-6.08	1.46	0.38-6.10	0.585
ALS Provider					
DOC/PARA combination	2.06	0.81-5.73	1.94	0.59-6.91	0.288
PARA	1.37	0.54-3.82	1.09	0.32-3.96	0.89

This table describes unadjusted and adjusted regression models for potential factors affecting the decision to place a cervical collar on patients with blunt cervical trauma.

OR = odds ratio; **AOR** = adjusted odds ratio; **CI** = confidence interval; **Bold** = statistically significant p value;

C-spine = cervical spine; **Military circumstances** = events include either trauma sustained during military operations or military training; **MVC** = motor vehicle collision; **ETI** = endotracheal intubation; **GCS** = Glasgow coma scale; **AVPU** = alert, response to vocal stimulation, response to pain, unresponsive; **VAS** = visual analogue scale (for pain); **SPO2** = peripheral capillary oxygen saturation; **ALS** = advanced life support; **DOC** = doctor, **PARA** = paramedic;
