# Supplementary figures and images for: Lack of Association between Cervical Spine Injuries and Prehospital Immobilization: From Tradition to Evidence
Source: J Clin Med. 2024 Aug 18;13(16):4868. doi: 10.3390/jcm13164868 (PMC11355150; doi:10.3390/jcm13164868)

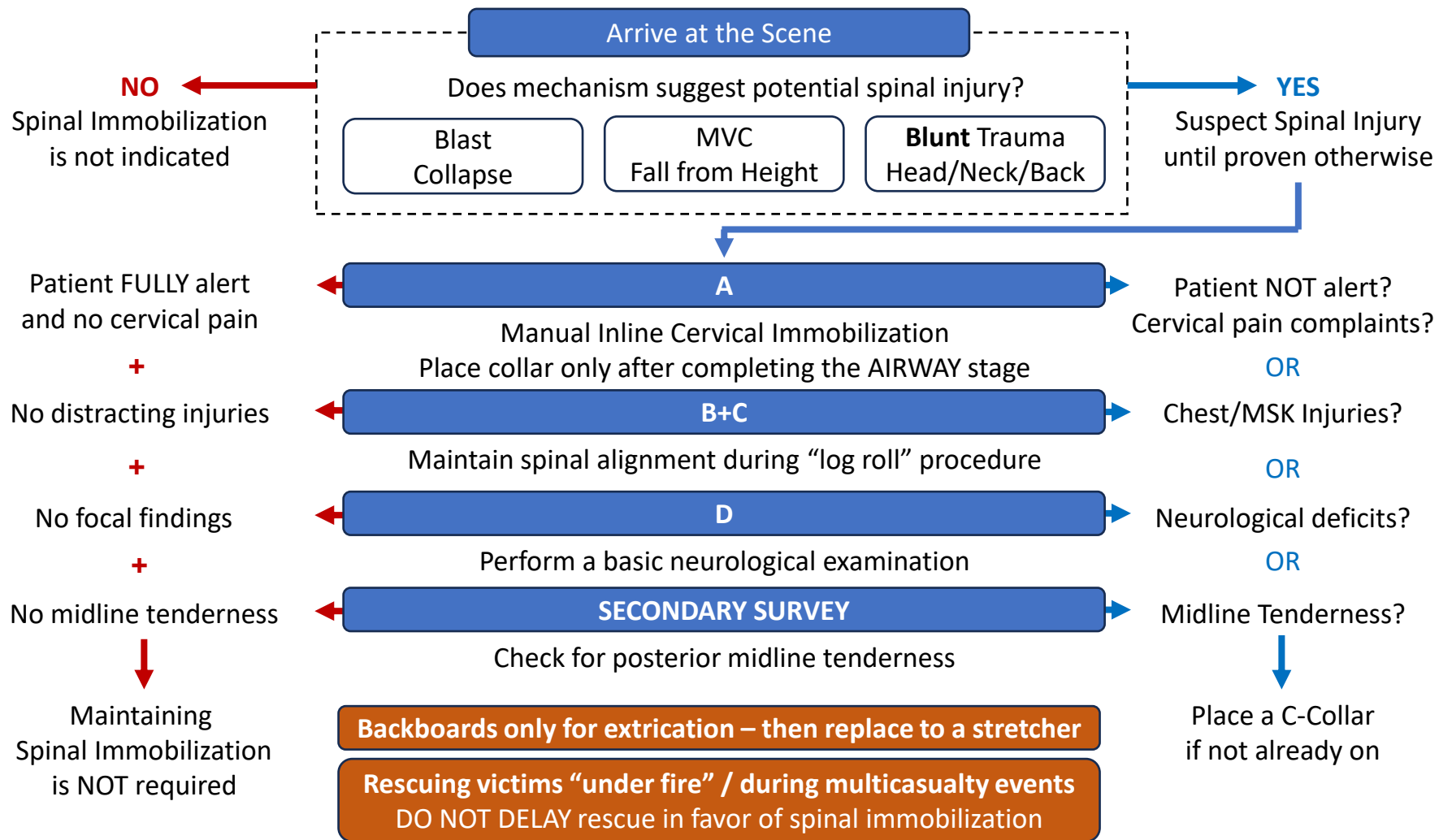

Supplement: Supplementary file 1 [file jcm-13-04868-s001.zip › Supplemental Figure S1.pdf]
